# Neural field simulator: fast computation and 3D-visualization

**DOI:** 10.1186/1471-2202-14-S1-P179

**Published:** 2013-07-08

**Authors:** Eric J Nichols, Axel Hutt

**Affiliations:** 1 INRIA CR Nancy - Grand Est, Equipe NEUROSYS, 615 rue du Jardin Botanique, 54602 Villers-les-Nancy, France

## 

This work presents a simulator that facilitates dynamic neural field (DNF) calculations obeying models of the type

$$\frac{\partial V\left(x,t\right)}{\partial t}=-V\left(x,t\right)+\int K\left(x-y\right)S\left[V\left(y,t-\left|x-y\right|/c\right)\right]dy+I\left(x,t\right)$$

involving axonal finite transmission speed c. The underlying numerical computation method [1] utilizes a Fast Fourier Transform in space. Motivation for the work arises from a need for a visualization tool that is useful to the largest number of DNF researchers, allows for the tailoring of code and has fast while visually appealing output. The simulator can operate on all major operating systems and the wxWindows library is used to provide a native cross-platform look and feel. It is open source and enables researchers to modify the simulator in any beneficial way. Output of data in 3 dimensions is provided by PyOpenGL which brings the speed and graphical detail of low-level OpenGL to the agile Python language.

**Figure 1:**
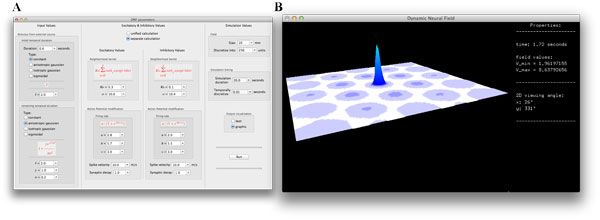
**The interface to choose neural field parameters is shown in panel A**. The graphical view of a field's voltage is displayed in panel **B**

The simulator consists of a window where one can choose DNF parameters and the output format. The parameter window (Figure 1A) consists of three sections. The first allows the user to choose field (size and discretization) and input parameters. The second section gives the user the option of calculating excitatory and inhibitory activity separately or in a unified manner. In this section, the user can choose the field's spatial connectivity kernel and the nonlinear transfer function, transmission speed, and synaptic decay time constant. Selection of simulation values such as duration, temporal discretization and output visualization are offered in the third section. The selected values in this window are saved for quick subsequent modification of particular parameters.

The options for output include text based notifications and graphical visualization in 3 dimensions (Figure 1B). These output methods allow the user to pause and modify variables during the DNF's calculations. Moreover, the user is able to make a video of the animated field for later review.
